# SHA-256 Hardware Proposal for IoT Devices in the Blockchain Context

**DOI:** 10.3390/s24123908

**Published:** 2024-06-17

**Authors:** Carlos E. B. Santos, Lucileide M. D. da Silva, Matheus F. Torquato, Sérgio N. Silva, Marcelo A. C. Fernandes

**Affiliations:** 1 InovAI Lab, nPITI/IMD, Federal University of Rio Grande do Norte (UFRN), Natal 59078-970, Brazil; ceduardobsantos@gmail.com (C.E.B.S.J.); lucileide.dantas@ifrn.edu.br (L.M.D.d.S.);; 2 Leading Advanced Technologies Center of Excellence (LANCE), nPITI/IMD, UFRN, Natal 59078-970, Brazil; 3 Federal Institute of Education, Science and Technology of Rio Grande do Norte, Santa Cruz 59200-000, Brazil; 4 Department of Computer Engineering and Automation, Federal University of Rio Grande do Norte, Natal 59078-970, Brazil

**Keywords:** FPGA, IoT, blockchain, SHA-256, hardware

## Abstract

This work proposes an implementation of the SHA-256, the most common blockchain hash algorithm, on a field-programmable gate array (FPGA) to improve processing capacity and power saving in Internet of Things (IoT) devices to solve security and privacy issues. This implementation presents a different approach than other papers in the literature, using clustered cores executing the SHA-256 algorithm in parallel. Details about the proposed architecture and an analysis of the resources used by the FPGA are presented. The implementation achieved a throughput of approximately 1.4 Gbps for 16 cores on a single FPGA. Furthermore, it saved dynamic power, using almost 1000 times less compared to previous works in the literature, making this proposal suitable for practical problems for IoT devices in blockchain environments. The target FPGA used was the Xilinx Virtex 6 xc6vlx240t-1ff1156.

## 1. Introduction

The development of new data transmission and connectivity methodologies necessitates enhanced information security control to ensure the transmitted information’s confidentiality, integrity, and availability [1]. Technologies and tools that meet these requirements and deliver performance commensurate with the processing load are indispensable today. For instance, blockchain technology enhances these methods and supports decentralized information, privacy, and reliability. Information security management may include integrity verification using a hash-based message authentication code (HMAC), digital signature implementation via public key infrastructure (PKI), and data transmission over the Transport Layer Security (TLS) protocol. Power consumption, hardware size, and processing time are critical factors in developing Internet of Things (IoT) solutions [2]. The work presented in [3] discusses Internet Protocol Security (IPSec) and the need for rapid solutions that leverage high network connection speeds, as provided by optical fibers, exceeding 30 Gbps.

FPGA technology was chosen for this project to enhance performance and power efficiency in IoT devices for blockchain applications due to its high throughput, low power consumption, and flexible architecture [4]. FPGAs are reconfigurable hardware platforms consisting of thousands of logic cells, which function as dedicated hardware for specific algorithms following a synthesis process. These devices are crucial in developing specialized hardware, offering performance on par with ASIC implementations but with reduced development time [5]. Various studies in the literature have utilized FPGAs as a development platform to accelerate complex algorithms [6,7,8,9,10] and to enhance blockchain applications in IoT contexts [11,12,13,14,15,16,17,18,19].

This work proposes dedicated hardware using FPGA for the SHA-2 family algorithm (SHA-256). The proposed hardware employs a 256-bit fixed-length hash, widely utilized in methodologies and protocols such as proof of work (one of the blockchain consensus algorithms), Merkle tree [13], HMAC, PKI, TLS, IPSec [20], and PGP, SSH, S/MIME [21]. The rationale for using FPGA hardware is twofold: to accelerate blockchain operations and enhance IoT devices’ security within blockchain environments. By leveraging the parallel processing capabilities of FPGA, our implementation significantly increases the throughput of the SHA-256 hashing process, which is critical for blockchain operations such as mining and transaction verification. Additionally, dedicated hardware secures these operations against various attacks, making the overall system more robust and reliable.

We present the hardware implementation on an FPGA, highlighting remarkable results regarding the balance between hardware resources, throughput, and power consumption using multiple cores for the SHA-256 algorithm. This proposal is particularly suitable for practical challenges in blockchain environments for IoT devices [22,23,24], achieving a throughput of approximately 1.4 Gbps with 16 cores on a single FPGA and reducing dynamic power consumption by nearly ×1000 compared to existing works. The chosen device for validating the proposal was the Xilinx Virtex 6 xc6vlx240t-1ff1156 FPGA.

While many studies have explored the use of hardware to accelerate blockchain operations, practical integration with blockchain protocols is often lacking. Our work addresses this gap by demonstrating a concrete use case where our FPGA-based SHA-256 implementation is integrated into a blockchain protocol designed for IoT environments. The main contributions of this work are as follows:

- Enhanced processing capacity: By utilizing a multi-core architecture on the FPGA, the proposed implementation significantly improves the processing capacity of IoT devices, achieving a throughput of approximately 1.4 Gbps for 16 cores on a single FPGA.
- Energy efficiency: The FPGA implementation demonstrates substantial power savings, reducing dynamic power consumption by nearly 1000 times compared to existing solutions, making it suitable for power-constrained IoT applications.
- Security and privacy: Integrating the SHA-256 algorithm into IoT devices enhances data integrity and confidentiality, addressing critical security and privacy concerns in IoT communications.
- Scalability and flexibility: The FPGA-based approach provides a flexible and scalable solution that can be adapted to various IoT scenarios, including smart cities, the Industrial IoT, and real-time healthcare monitoring systems.

This paper is structured as follows. Section 2 presents previous work on implementing SHA-256 on FPGA solutions. Section 3 is divided into three subsections that introduce the background of the blockchain (Section 3.1), the IoT in a blockchain context (Section 3.2), and a subsection on the SHA-256 algorithm (Section 4), highlighting some details. In Section 5, the proposed design is explained in depth. The implementation results and comparisons with previous work are provided in Section 6. Finally, Section 7 reports the conclusions of this work.

## 2. Related Work

The development and implementation of secure and efficient hashing algorithms in hardware, particularly in FPGA, have gained significant attention in recent years due to their critical role in enhancing the performance and security of IoT devices. Several surveys have highlighted the advancements and challenges in this area, providing a comprehensive overview of the current state of research and future directions. Recent survey articles from high-impact journals, such as [25,26,27,28,29], have extensively reviewed the implementation strategies, optimization techniques, and performance metrics of cryptographic algorithms on reconfigurable hardware platforms.

The first published implementation of the SHA-256 algorithm on an FPGA was by [30], utilizing the Pilchard development kit with a Xilinx Virtex XCV300E-8 FPGA. This design employed several shift registers in series, segmented into three blocks to manage the variables associated with the SHA-256 algorithm. The initial block, the message scheduler, consists of sixteen 32-bit shift registers arranged serially to handle the entire 512-bit initial message. The subsequent block, the compression function, is similarly constructed using serialized shift registers. The final block comprises eight 32-bit registers that store the hash variables. This implementation contrasts with the parallelized approach of the current proposal, which eschews the serialized method. Ultimately, this setup achieved an 87 Mbps throughput with an 88 MHz clock, utilizing 1261 slices from a Xilinx FPGA.

The work presented by [31] employs a Xilinx Virtex v200pq240 FPGA to implement a unified architecture for three distinct algorithms from the SHA-2 family: SHA-256, SHA-384, and SHA-512. This implementation features a singular module that executes all required iterations for each algorithm using a rolling loop mechanism—akin to the implementation described herein, albeit restricted to the SHA-256 algorithm alone. The output from this module interacts with the values of the algorithm’s initial constants stored in ROM, culminating in a module dedicated to storing the hash code. The SHA-256 implementation, requiring 65 clock cycles for completion, utilized 2,384 configurable logic blocks (CLBs), achieved a maximum frequency of 74 MHz, and delivered a throughput of 291 Mbps.

Ref. [3] focused on the high performance of the SHA-256 algorithm, utilizing a central structure segmented into four parts and arranged in a pipeline architecture. This study incorporated several performance enhancement techniques, including retiming, precomputation, and loop unrolling, significantly improving throughput and the throughput-to-area ratio. Contrary to the implementations discussed in this document (Section 5), the paper did not employ a pipeline architecture or the improvement above techniques. Instead, a multiple core approach was utilized. Through this methodology, thr authors managed to compute four hash values within 32 clocks, allocating 8 clocks to each pipeline segment. The experiments utilized Xilinx Virtex FPGAs models 5 and 6, with the most effective results achieved on the Virtex 6, recording a frequency of 172 MHz and a remarkable throughput of 11,008 Gbps using 1831 slices.

In another study, Ref. [32] proposed a compact SHA-256 solution for mobile devices. This implementation capitalized on the reuse of modules, with its principal component being an arithmetic logical unit (ALU) encompassing four entries, referred to as the Hash ALU. This architecture required 280 clocks to generate a single hash, utilizing merely 139 slices and 527 lookup tables (LUTs). It achieved a frequency of 64.45 MHz and a throughput of 117.85 Mbps, using a Xilinx Virtex 5 FPGA. The approach proposed in the current document also reuses modules to facilitate the sixty-four hashing rounds but does so without implementing the ALU concept.

The researchers in [33] developed a pipeline register architecture on a Xilinx Virtex-4 FPGA, similar to that described in [3]. This architecture was divided into two main components: the expander and the compressor. Employing this method, the system achieved a maximum frequency of 170.75 MHz and a throughput of 1344.98 Mbps.

In contrast, the study in [34] sought to create a high-speed hardware implementation for the SHA-256 algorithm. This effort resulted in two versions, termed SHA-256 and SHA-256 unfolding. The design utilized six modules, mirroring the approach proposed here but with distinct differences in the input handling and module arrangement within the FPGA. The unfolding version from [34] required only 32 clock cycles, used 1215 lookup tables (LUTs), 871 registers, and achieved a throughput of 2429.52 Mbps on an Arria II Gx FPGA from Altera.

Furthermore, Ref. [21] introduced fully pipelined, area-efficient architectures for SHA-1 and SHA-256 using block random access memory (BRAM), implemented across various Xilinx FPGA families (Virtex-4, Virtex-5, Virtex-6, and Kintex-7). A vital aspect of these designs was the strategic placement of BRAMs to minimize the use of registers. The SHA-256 architecture on a Virtex-6 FPGA reached a frequency of 276.4 MHz, utilized 11,660 slices and 35 BRAMs, and achieved a remarkable throughput of 141.517 Gbps. This implementation uses BRAM modules, which contributed to achieving the highest throughput among the discussed references.

The research presented by [35] focused on developing a system-on-a-chip (SoC) design based on a processor and hardware accelerator for the SHA-256 hash algorithm using the high-level synthesis (HLS) method on a Xilinx ZC 702 board. The primary objectives of this study were to minimize hardware resource utilization, processing time, and power consumption. The authors proposed three distinct solutions: the first was purely software-based, utilizing the ARM Cortex A9 processor without any optimizations, achieving 399 slices, five BRAMs, 1322 flip-flops, a frequency of 222 MHz, and a throughput of 96 Mbps. In the second solution, the UNROLL pragma was applied to external loops of the compression function equations, significantly reducing latency and enhancing architectural speedup. The third solution incorporated an AXI4-Stream bus and AXI4-Lite control at the input/output of the top-level function. The synthesis report for this implementation on an XC7Z020 FPGA showed 21,197 LUTs, two BRAMs, and 19,212 flip-flops, with a maximum frequency of 175 MHz. This process differs from that proposed here as it utilizes BRAM, ARM Cortex, and AXI4-Lite control, whereas our implementation is exclusively FPGA-based and employs the rolling loop concept.

Further developments in SHA-256 designs focused on reducing the critical path via rescheduling, as detailed in [36]. This approach involved the creation of variables for pre-calculation prior to the main loop, allowing the round computations to be divided into two pipeline stages. The hardware configuration for SHA-256 included a carry-save adder (CSA) and the addition of four operands implemented using a 4-2 compressor and an adder. The authors utilized six registers to store the variables A, B, C, E, F, and G, mirroring the structure used in this paper (Section 5), but without incorporating pipeline stages or precalculated variables. The SHA-256 implementation described by [36] achieved a throughput of 1.984 Mbps, used 979 slices, operated at a frequency of 255.7 MHz, and required 66 clock cycles. This design was implemented on a Xilinx Virtex-4 XC4VLX100-12 FPGA using the Xilinx ISE 14.7 tool.

Ref. [37] discusses the implementation of the SHA-256 algorithm in both hardware and software for embedded applications on a Zynq 7000-based FPGA using the XC7Z020 chip. The authors highlight several advantages of high-level synthesis (HLS) over low-level synthesis (LLS), such as faster development times and greater adaptability. They assert that HLS allows for early estimation of area cost, frequency, and latency despite some limitations in converting HLS code to register transfer level (RTL). According to the study, the hardware/software (HW/SW) integration approach, which combines a microprocessor system with programmable logic on the same chip, is deemed optimal. The hardware-only component of their implementation utilized 32-bit registers, ROM, and logical operations, achieving a throughput of 1063 Mbps, a frequency of 135 MHz, and 1305 slices.

In related research, Ref. [38] proposed an architecture designed to optimize metrics such as throughput, frequency, and power consumption, which aligns closely with the objectives of this current work. Their architecture comprises four main components, controller, communication, extension, and compression, configured into two pipeline stages. The first stage extends the message from 16 to 64 words, and the second involves the extension and compression modules. Unlike other designs and the one discussed in this paper, they implemented a finite-state machine (FSM) using shift registers for stage transitions, which they claim reduces hardware usage and overall power consumption significantly. Their results, obtained on a Xilinx Artix-7 (xc7a200t), showed a consumption of 1310 lookup tables (LUTs), 881 registers, and 327 slices, achieving a maximum frequency of 141.84 MHz and a throughput of 1404 Mbps. This design, while achieving a similar throughput to that proposed here (see Section 6), operates at a higher frequency but uses fewer slices.

The study presented in [39] utilizes OpenCL to implement the SHA-256 algorithm on an FPGA, applying eight different optimization techniques, including the insertion of local memories, loop splitting, loop unrolling, and loop pipelining. According to the authors, OpenCL is divided into host code and kernel configuration. They conducted experiments using a Nallatech 510T board equipped with two Arria 10 1150 GX FPGAs installed on a Dell machine via a PCIe interface. The best baseline performance using the VSUP kernel required more resources than the other variants due to the intensive use of loop unrolling optimization. Nonetheless, the results achieved were a throughput of 3973 Mbps and a clock frequency of 179.4 MHz.

When researching the use of blockchain for IoT devices, we can find several papers that discuss applications in various industrial sectors, as presented by [13,14,15,16,17,18,19,40,41]. These papers explore different methods for these devices to authenticate themselves on the blockchain network. For example, the study in [40] proposes the CBcA schema, a device authentication mechanism during the block validation phase, to prevent intrusions and data tampering. The CBcA schema is divided into two stages: the first is the registration phase of the IoT device, using a certification authority (CA) and a verifier control center (VCC), which employs the concept of digital signatures and public–private key pairs and links the device ID records to the created keys; following this process, the blockchain block is generated. The second stage involves the transmission of this block after calculating the hash of the current block and the previous block, both utilizing the SHA-256 algorithm for this purpose.

There is also an approach in the registration and authentication process for IoT devices applied to smart cities, as described in [42]. The paper highlights several security issues that blockchain usage can address, such as distributed denial-of-service (DDoS) attacks, outdated firmware, and environments with unknown devices. The proposal develops an API gateway to enable IoT devices to sign, identify, and authorize the transmission of messages using the Ethereum blockchain, with smart contracts and the Merkle tree. In the literature review, the authors discuss fourteen different papers that cover the IoT and blockchain in various approaches and contributions, demonstrating the feasibility and significance of this theme in the evolution of various applications.

The healthcare sector is a domain where sensitive personal information is constantly handled, necessitating a high degree of information security measures. In this regard, Ref. [43] proposes an IoT architecture based on blockchain to enhance health data security using an identity-based encryption (IBE) algorithm. This paper utilizes Ethereum as the blockchain platform and the SHA-256 algorithm to compute the Merkle tree. Other research, such as that by [44], also discusses the use of blockchain and the IoT in healthcare to preserve the privacy of patient information using block transactions. Each transaction is hashed using SHA-256, including at the end of the block formation, along with the timestamp.

The studies presented in [11,12] provide insights into the use of FPGA within the blockchain context for the IoT. Both feature similar structures in which they discuss the operation of blockchain, FPGA, and the SHA-256 algorithm. They also use Verilog HDL and ModelSim to simulate an “ABC” input message (616263 in hexadecimal) synthesized on a Xilinx Artix-7 low-voltage XC7A200TL-ffg1156 FPGA. The study in [12] details the use of 513 flip-flops and 133 LUTs at a frequency of 101.672 MHz but does not report the throughput. Meanwhile, the work in [11] achieved a throughput of 1190.6 Mbps using 2282 slices, 7174 LUTs, and a frequency of 302.186 MHz.

The work presented in [45] detailed analysis of the performance of hardware caching techniques for blockchain databases, focusing on using FPGAs to enhance data access efficiency in blockchain systems. The research specifically addresses the storage of key–value pairs, a common mechanism in full blockchain nodes that face scalability and response time challenges due to high user request volumes. The authors implement and compare different caching techniques, such as direct mapping and 2-way and 4-way associative techniques, analyzing hit and miss rates under various conditions. They conclude that associative techniques offer better hit rates than direct mapping, especially in larger datasets, highlighting the importance of choosing the appropriate caching technique to optimize performance and scalability in practical blockchain applications.

The work presented in [24] discusses the implementation of blockchain consensus algorithms on FPGAs, focusing on optimizing performance, security, and scalability for IoT applications. The authors explore different consensus methods, such as proof of work (PoW), proof of stake (PoS), and proof of authority (PoA), using the VHDL language for programming the FPGAs. The study aims to demonstrate the importance of optimizing the execution time of consensus through intellectual properties (IPs) in VHDL, assessing their impacts on security and efficiency. Additionally, it compares the resource consumption, execution time, and energy efficiency of these algorithms, providing a crucial reference for selecting appropriate consensus mechanisms for embedded systems.

The work presented in [46] discusses the implementation of a consortium blockchain system called HPCchain, designed explicitly for authentication in the Industrial Internet of Things (IIoT) using a combination of CPU and FPGA-based physical unclonable functions (PUFs). This system aims to address device heterogeneity and scalability issues in the IIoT, leveraging the unique characteristics of devices to ensure secure and reliable authentications. HPCchain incorporates a PUF-based consensus mechanism that enhances security and reduces system resource demands through a PUF-empowered credit scheme, which assesses the historical behavior of devices on the network. Extensive experiments show that HPCchain can effectively integrate various IIoT devices without performance loss, providing robust authentication and improved system throughput.

This paper presents significant advancements over the works discussed in [24,45,46], addressing challenges related to energy efficiency and the scalability of blockchain systems in IIoT applications. While the study in [45] focuses on performance analysis of hardware caching techniques for blockchain databases, this paper extends the scope to integrate energy-efficient solutions and adaptable consensus mechanisms critical for the IIoT. The work in [24] explores the implementation of consensus algorithms on FPGAs. However, our work goes further by providing a deeper and more optimized integration with the specific hardware and software requirements of the IIoT. Furthermore, the study in [46] introduces a consortium blockchain system for authentication in the IIoT, using hybrid CPU-FPGA PUFs. However, our paper stands out with its innovative approach to dynamic consensus management that adapts to real-time load and operational changes. This is a crucial need for IIoT environments facing variable and unpredictable operating conditions. This functionality enhances energy and operational efficiency and ensures high availability and reliability.

## 3. Blockchain in the IoT

IoT devices have grown considerably with the advancement of wireless network connections and the widespread adoption of technologies such as 5G. However, these devices often need to rely on each other without proper authentication and authorization methods [42]. Therefore, the implementation of blockchain technology is becoming increasingly viable. This is particularly true for edge computing devices, which process sensor data locally before sending it to the cloud or a gateway [42]. To better understand the proposal presented in this section, we describe some of the fundamental concepts of blockchain and how it relates to the IoT.

### 3.1. Blockchain Overview

Blockchain is a distributed ledger technology (DLT) designed to be tamper-resistant [47]. Despite its prevalent use in the financial market, its applications are not limited to this sector alone. The interest in blockchain stems from its unique characteristics for data (or transaction) storage and the validation of these data through a chain of blocks in a completely decentralized manner. This validation is achieved through verification chains so that if any data are modified, the entire blockchain network will be aware [40]. This process utilizes cryptographic fundamentals such as hash functions, symmetric cryptography, asymmetric cryptography, and Merkle trees [47].

The term “blockchain” originated from the first paper that described this concept, authored by “Satoshi Nakamoto” [48]. This paper provides a detailed understanding of the first peer-to-peer payment system using blockchain technology via Bitcoin. It also explains the necessity of a consensus algorithm through the computation of SHA-256 hash algorithms (as in the case of Bitcoin’s proof of work) to verify transactions that have occurred and are legitimate, thereby validating a new block in the chain. Furthermore, transactions must be explicitly disseminated to various network nodes, enabling these nodes to validate the legitimacy of transactions in previous blocks.

**Figure 1:**
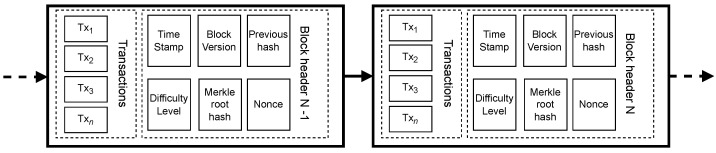
General scheme diagram of blockchain.

Figure 1 presents an example of a block structure in a generic blockchain. A block is divided into two parts: one for the transactions and the other for the header. The number of transactions varies per block, ranging from dozens to hundreds depending on the blockchain technology used; these numbers are represented by $Tx_{1}$ to $Tx_{n}$ in Figure 1. The header records the information about the block and its history in the chain. Thus, it is divided into the following fields: previous hash, which carries the hash of the entire originating block (block N−1) for the current block (block *N*), thereby maintaining the order of the blocks within the chain; the block version indicates a set of rules for block validation; the Merkle tree contains a hash of the root of the Merkle tree of this block’s transactions; the difficulty level represents the effort required to mine the block; the nonce is a random value determined by miners to solve the consensus algorithm problem; and the timestamp records the creation time of the current block [49].

**Figure 2:**
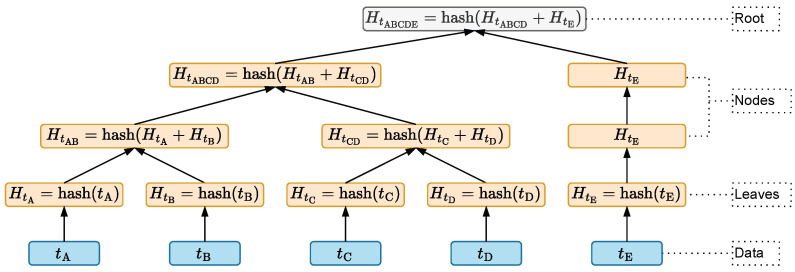
Merkle tree structure.

A block added to a blockchain undergoes a process called mining, which involves validating the transactions and the structure of the header. However, to be mined, a block must contain transactions (or data) tied to that block. These transactions are sent through the nodes of the blockchain’s peer-to-peer network. Storing all the transactions from multiple blocks would lead to high storage costs for blockchain technology; thus, to address this, a summary of all the transactions within the block is created using a Merkle tree, as found in the block’s header shown in Figure 1. A Merkle tree is a data structure used to summarize and verify the integrity of a large dataset using hash functions [49]. There are various types of hash algorithms; however, SHA-256 is commonly used, as mentioned in [42,48,50,51]. Figure 2 illustrates the organization of the tree, with the root hash $R=H_{ABCDE}$ at the top, the hashes of the nodes immediately below, which receive the hashes of the data or transactions ($t_{A}$, $t_{B}$, $t_{C}$, $t_{D}$, $t_{E}$), are displayed on each branch by the respective hashes ($H_{t_{A}}$, $H_{t_{B}}$, $H_{t_{C}}$, $H_{t_{D}}$, and $H_{t_{E}}$), and calculated by concatenating them in pairs or singly if the total number of transactions per block is odd.

### 3.2. Blockchain in the IoT

In the context of the IoT, there is a wide variety of applicability for using blockchain to strengthen the information security triad (confidentiality, integrity, and availability) in scenarios involving devices with low reliability [47]. According to [43], the IoT and blockchain are emerging areas in the information technology (IT) sector, and as such, they warrant continued attention and research enhancements.

One of the main functions that blockchain has addressed in networks with various IoT devices relates to the unreliability of the transmitted data. Moreover, these data (originating from sensors connected to such devices), in a traditional operation without blockchain, are generally stored in a single database, i.e., a single point of failure [42,52], vulnerable to distributed denial-of-service (DDoS) attacks and infrastructure problems. There is also the possibility of alteration in the base of the collected data without the actual users, the consumers of the information, being aware of these adulterations. Additionally, there is the potential for devices to enter the network and send data that do not correspond to reality or flood the network. A third party must be needed to validate the transactions.

**Figure 3:**
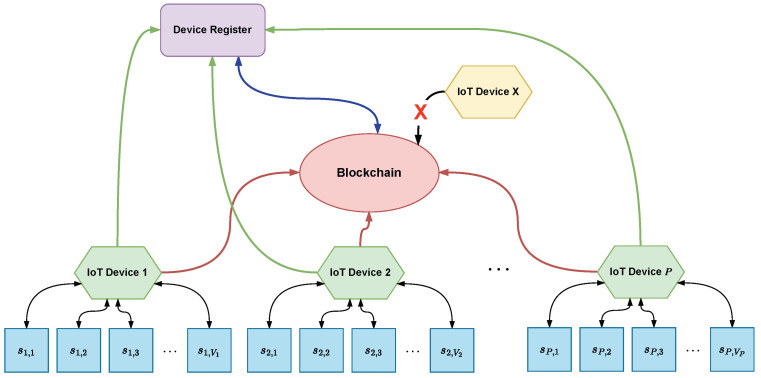
Blockchain-based IoT network.

Figure 3 illustrates an example of blockchain technology application in a network of *P* IoT devices, where each *p*-th device is connected to $V_{p}$ sensors, with $s_{p,v}$ representing the *v*-th sensor connected to the *p*-th device. Figure 3 depicts a device registrar and the blockchain itself. The figure shows *P* IoT devices (in green), which are part of a valid network, their sensors (in blue), and one unauthenticated device (in yellow). The initial stage of communication occurs when the IoT devices are registered with a registrar, as shown in Figure 3 by the green arrows. Consequently, only authenticated IoT devices can participate in the blockchain. An authentication and authorization process is essential for these devices to transmit transactions on the blockchain. This process serves as a verification mechanism to ensure that only previously registered IoT devices can share blocks and transactions, thus preventing maliciously added devices (depicted in yellow) from launching attacks or otherwise disrupting the proper use of the technology. The blue arrow represents this process in Figure 3. For example, as discussed in [42], a blockchain API gateway is employed for these authentication and authorization functions to facilitate secure interactions with the blockchain. In contrast, [40] utilizes a certification authority (CA) and a verifier control center (VCC) for similar purposes. Furthermore, Ref. [53] describes the use of a certification center to certify the regional nodes (similar to the IoT devices shown in Figure 3) and manufacturers, with each regional node maintaining comprehensive records about the devices, manufacturers, and their permissions within the blockchain.

The red arrows in Figure 3 depict when IoT devices send information to the blockchain. The nature of this information varies according to the blockchain proposal presented; for example, in [47], there is a sequence of messages divided into three layers (IoT et al. layer), which provide information regarding the authentication method of the devices, transaction data, encryption keys, among others. Similarly, Ref. [40] proposes in the architecture that only after authenticating the devices (green arrows) will it be possible to participate in the blockchain. In both cases, participation in the blockchain occurs when the sensor data $S_{n}$ are stored as transactions $Tx_{n}$ and propagated via broadcast, where all devices receive and initiate the process of forming a block (Figure 1) to be added to the blockchain. This stage is called mining, when the problem related to the consensus algorithm is resolved.

When a device successfully resolves the problem, it announces the newly created block to the entire network and adds it to the chain. After this, mining a new block begins to validate the newly added block, and consequently, the transactions. To prevent IoT devices from storing all transactions and facing related issues, Merkle trees are used, thus enabling the validation of transactions through hashes, as shown in Figure 2.

Thus, the SHA-256 algorithm is quite prevalent in blockchain technologies, whether in the consensus algorithm, the creation of the Merkle tree root, or the authentication process with the CA. Therefore, in the subsection below, we describe how this algorithm works.

## 4. SHA-256 Algorithm Description

Algorithm 1 shows the SHA-256 pseudocode used as a reference for the hardware implementation proposed in this work.

The input of Algorithm 1 is the input message expressed by

(1) $$m_{i}=\left[\begin{array}{cccc} m_{0} & m_{1} & \ldots & m_{K_{i}-1} \end{array}\right],\;m_{k}\;\in \;\left\{0,1\right\}\;\forall \;k$$

where $m_{i}$ represents the input message where $K_{i}$ is the arbitrary bit size of the message. The message ($m_{i}$) goes through two extension processes, in order to ensure that at the end of them $m_{i}$ plus the extension result has a length divisible by 512 [54]. The extension processes are represented in lines 1–5 from Algorithm 1. The first is a padding process (line 2) that adds the binary 1(one) to identify the end of the message and then completes with binaries 0(zero) until the new array, identified as $z_{i}$, reaches a length of 448 bits or a multiple of 512 plus 448 bits (line 3). Bits added from the padding process are identified as $p_{i}$, where $K_{i}+p_{i}=448\;\mathrm{mod}\;512$. The second extension process is called parsing (line 4) and consists of adding another 64 bits that contain information about the size of the original $m_{i}$ message in a binary representation. The result of the parsing processing is identified as $v_{i}$. The vector $z_{i}$ will be updated with the result of both extension processes, composed of the original message and the bits added in the padding and parsing processes (line 5).
**Algorithm 1** SHA-256 for each *i*-th message $m_{i}$1:$z_{i}\leftarrow \left[m_{i}\right]$2:$p_{i}\leftarrow \mathrm{Padding}\left(K_{i}\right)$3:$z_{i}\leftarrow \left[m_{i}\;p_{i}\right]$4:$v_{i}\leftarrow \mathrm{Parsing}\left(K_{i}\right)$5:$z_{i}\leftarrow \left[m_{i}\;p_{i}\;v_{i}\right]$6:$h_{i}\leftarrow \mathrm{HashInitialization}\left(\;\right)$7:**for** j←0 **to** $L_{i}-1$ **do**8:   $b_{j}\leftarrow \mathrm{MessageSplit}\left(z_{i}\right)$9:   n←−110:   $\mathrm{WH}\left(n\right)\leftarrow h_{i}$11:   **for** n←0 **to** 63 **do**12:     $s0\left(n\right)\leftarrow S0\mathrm{FunctionCalculation}\left(n,b_{j}\right)$13:     $s1\left(n\right)\leftarrow S1\mathrm{FunctionCalculation}\left(n,b_{j}\right)$14:     $w\left(n\right)\leftarrow \mathrm{WFunctionCalculation}\left(n,b_{j},s0,s1\right)$15:   **end for**16:   **for** n←0 **to** 63 **do**17:     S1(n)←S1FunctionCalculation(n,E(n))18:     S0(n)←S0FunctionCalculation(n,A(n))19:     maj(n)←MajFunctionCalculation(n,A(n),B(n),C(n))20:     Ch(n)←ChS0FunctionCalculation(n,E(n),F(n),G(n))21:     WH(n)←HashVariablesUpdate(H(n))22:   **end for**23:   $h_{i}\leftarrow \mathrm{HashUpdate}\left(\mathrm{WH}\left(n\right)\right)$24:**end for**

After the steps of the extension process, the initialization of the hash values variables occurs (line 6). The initial hash values are a vector with 8 elements of 32 bits, here represented as ha through hh, and their values are obtained by the first 32 bits of the fractional parts of the square roots of the first eight prime numbers [55]. This fixed hash number C = 256 bits is the union of all variables. The hash values are initialized to $h_{i}$, which can be expressed by

(2) $$h_{i}=\left[\begin{array}{cccccccc} \mathrm{ha} & \mathrm{hb} & \mathrm{hc} & \mathrm{hd} & \mathrm{he} & \mathrm{hf} & \mathrm{hg} & \mathrm{hh} \end{array}\right].$$

The next step is the split of $z_{i}$ into $L_{i}$ 512-bit chunks, defined in the SplitMessage step (line 8 of Algorithm 1), where each chunk is stored in a vector $b_{j}$, divided into 16 words, $u_{j}\left[k\right]$, represented as

(3) $$b_{j}=\left[\begin{array}{cccc} u_{j}\left[0\right] & u_{j}\left[1\right] & \ldots & u_{j}\left[15\right] \end{array}\right],$$

where $u_{j}\left[k\right]$ is a 32-bit message.

The hash value, $h_{i}$, is initialized to the working variables, represented as WH(n) (line 10) can be expressed by

(4) $$\mathrm{WH}\left(n\right)=\left[\begin{array}{cccccccc} A(n) & B(n) & C(n) & D(n) & E(n) & F(n) & G(n) & H(n) \end{array}\right].$$

The loop referenced in line 11 of Algorithm 1 calculates the logical functions related to the expansion process of the 16 initial words of the message ($mathb_{j}$) to 64 words. At the last of these functions the message will be identified as w(n).

The FIPS 180-4 [54] denominates this phase as the hash preprocessing, which in addition to computing w(n), computes the values of s0(n) and s1(n), defined in lines 12 e 13 of the Algorithm 1 and expressed by

(5) s0(n)=rr(w(n−15),7)⊕rr(w(n−15),18)⊕rs(w(n−15),3),

(6) s1(n)=rr(w(n−2),17)⊕rr(w(n−2),19)⊕rs(w(n−2),10).

In these equations, the operation ⊕ is the bitwise exclusive OR and rr(r,s) identifies the function rightrotate, expressed as

(7) rr(r,s)=(r≫s)∨(r≪(32−s)),

where ∨, ≪, and ≫ are OR, left-shift, and right-shift operations, respectively. The rs(r,s) is the bitwise shift to the right without rotation.

The function w(n) presented in line 14 of the Algorithm 1 has the role of expanding the message w(n) consisting of 16 words (32 bits each) into 64, adding another 48 words, according to

(8) w(n)=w(n−16)+s0(n)+w(n−16)+s1(n)

In the second loop (line 16), the functions related to the hash processing are performed, according to RFC 4634 and FIPS 180-4 [54]. For each *n*-th iteration of each *j*-th block $b_{j}\left(n\right)$, the logic functions S1, S0, Ch, and Maj are calculated from the values of the working variables A(n), B(n), C(n) and E(n), F(n), G(n), as described by

(9) S1(n)=rr(E(n−1),6)⊕rr(E(n−1),11)⊕rr(E(n−1),25),

(10) Ch(n)=(E(n−1)∧F(n−1))⊕(¬E(n−1)∧G(n−1)),

(11) S0(n)=rr(A(n−1),2)⊕rr(A(n−1),13)⊕rr(A(n−1),22),

(12) Maj(n)=(A(n−1)∧B(n−1))⊕(A(n−1)∧C(n−1))⊕(B(n−1)∧C(n−1)),

where ¬ and ∧ are the NOT and bitwise AND operators, respectively.

After this step, the values of the variables A(n) to H(n) are updated (line 21). The update of the hash variable is expressed by

(13) H(n)=G(n−1),

(14) G(n)=F(n−1),

(15) F(n)=E(n−1),

(16) E(n)=D(n−1)+Temp1(n−1),

(17) D(n)=C(n−1),

(18) C(n)=B(n−1),

(19) B(n)=A(n−1)

and

(20) A(n)=Temp1(n−1)+Temp2(n−1)

in which

(21) Temp1(n)=H(n−1)+s1(n−1)+Ch(n−1)+K(n−1)+w(n−1),

(22) Temp2(n)=S0(n−1)+Maj(n−1)

and K(n) is a vector containing the first 32 bits of the decimal parts of the cubic roots of the first 64 prime numbers [54].

At the end of the algorithm, the final value of the hash code is produced after 64 iterations and through the sum of the hash working variables A(n) to H(n) with the initial hash values, initially stored in the vector $h_{i}$. The vector $h_{i}$ can be expressed by

(23) ha=A(63)+ha,

(24) hb=B(63)+hb,

(25) hc=C(63)+hc,

(26) hd=D(63)+hd,

(27) he=E(63)+he,

(28) hf=F(63)+hf,

(29) hg=G(63)+hg,

and

(30) hh=H(63)+hh.

## 5. SHA-256 Implementation on Reconfigurable Hardware

Parallel structures, a novel approach in FPGA implementations, have proven to be remarkably effective. In our implementation of the SHA-256 algorithm, we applied multiple cores in parallel to independently generate hashes for various messages simultaneously. This unique technique, where multiple copies of the architecture are placed on a single FPGA, allows for the handling of threads in parallel. Each clock cycle generates more than one hash message, limited only by the maximum number of cores implemented. Importantly, this technique does not significantly affect the critical path, thus improving throughput as more hashes are delivered concurrently.

**Figure 4:**
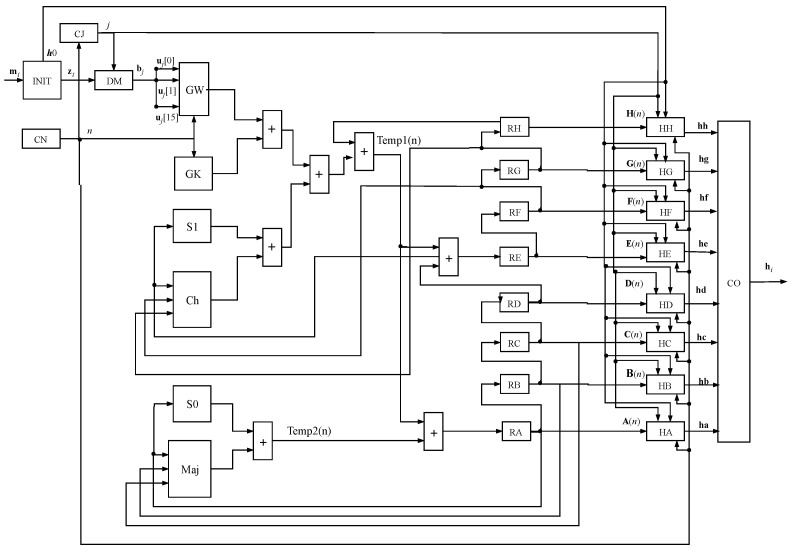
General architecture of the proposed SHA-256 hardware implementation.

Figure 4 details the implementation of a single core of the SHA-256 algorithm in hardware. For a multi-core implementation, the presented architecture is replicated according to the number of cores.

**Figure 5:**
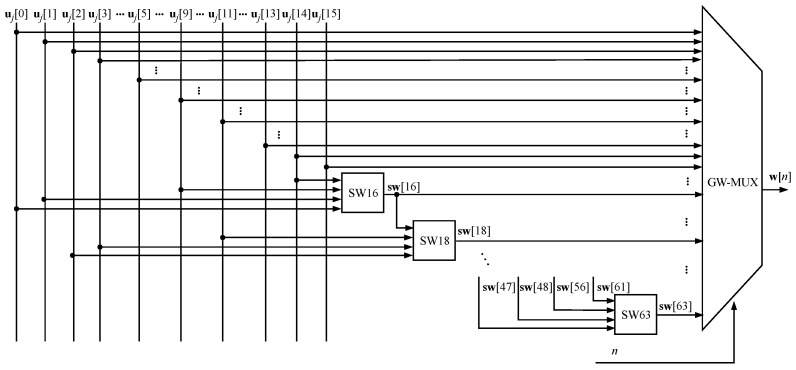
GW module architecture.

The design was developed to perform the operations presented in Algorithm 1. The first step in the signal flow occurs with the input of the *i*-th message $m_{i}$ in the INIT module, which executes the extension processes operations (lines 1–5) and hash initialization of the hash values (line 6) from Algorithm 1. The DM module performs the message split function, which divides the message into blocks, $b_{j}$ (line 8 from Algorithm 1), according to Equation (3). In turn, these blocks are split into 16 32-bit words, $u_{j}$, (Equation (3)), which are the inputs to the GW module, shown in Figure 5. This module is responsible for expanding the message w(n) to 64 words, expressed by Equation (8). Another input for both this module and the GK (which stores the values of the vector K(n)) is the output of the CN module, a 6-bit counter (ranging from 0 to 63) referring to the lines 11 and 16 of Algorithm 1. The CJ counter acts on the loop control described by line 7 of the same algorithm.

Furthermore, Figure 4 illustrates the direction of signals (or variables) among the datapath components, starting from the INIT module and moving through the registers RA, RB, RC, RD, RE, RF, RG, and RH. These signals represent the hash initialization function, as indicated in line 6 of Algorithm 1.

**Figure 6:**
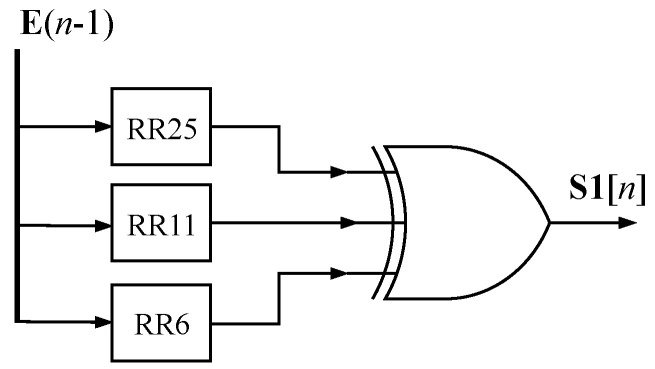
S1 module architecture.

The modules S1, Ch, S0, and Maj correspond to the implementations of Equations (9), (10), (11), and (12) respectively, which utilize 32-bit logic gates. In Figure 6, the implemented structure of the S1 module is visible, featuring a three-input XOR gate. The RR module can also be found in the figure, consisting of logic gates that perform the right-rotate operation, as described in Equation (7).

**Figure 7:**
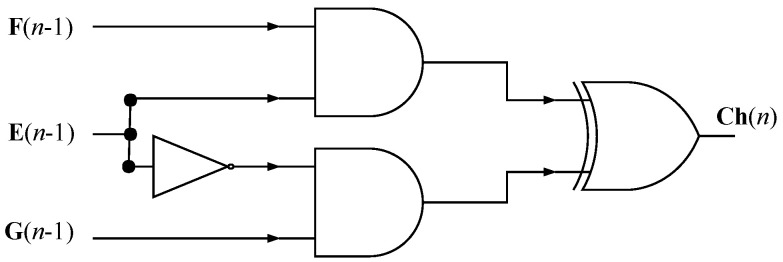
Ch module architecture.

In Figure 7, it is possible to visualize the structure of the Ch module that uses two AND logic gates, one XOR, and one inverter (NOT) (Equation (10)).

The values Temp1(n) and Temp2(n) are the results of the sum of the other modules (Equations (21) and (22)). Temp1(n) uses the value from the RH register too, which stores the WH(n) value of the vector containing the hashes’ variables, described by Equation (4). The process in Temp2(n) is similar to Temp1(n); however, it sums the S0 and Maj modules. Then, Temp1(n) added to Temp2(n) results in the value of the RA register after the first clock, which stores values in every interaction process with regards to the SHA-256 algorithm. Each register from RA to RH is updated in every clock within their 64 clocks to generate the hash code. The step of updating the hashes’ variables is performed on line 21 of Algorithm 1.

Hence, after 64 iterations from the for loop in *n* (line 16 of Algorithm 1), the parts constituting the hash code, ha, hb, hc, hd, he, hf, hg, and hh (Equation (2)), are updated by the modules HA, HB, HC, HD, HE, HF, HG, and HH, respectively, as per Equations (23) to (30). This step is performed in line 23 of Algorithm 1. Finally, in a further iteration, the module CO concatenates the eight 32-bit buses constituted by the signals ha, hb, hc, hd, he, hf, hg, and hh and produces a serial signal with the hash code $h_{i}$. The whole process takes 65 clocks.

## 6. Analysis and Results

This section presents a comprehensive analysis of our FPGA-based SHA-256 implementation, focusing on performance metrics such as throughput and power consumption. The results are compared with existing works to highlight the efficiency and advantages of our approach.

### 6.1. Hardware Validation

We perform the system validation by comparing the results obtained from the FPGA with known software implementations described in the literature in the *C* programming language [56] and from online (https://passwordsgenerator.net/sha256-hash-generator/ (accessed on 10 May 2024) presents an example of a verifier). The inputs consist of dozens of random words with sizes ranging between 4 and 8 characters. The generated hashes were identical in both the hardware and software implementations.

Table 1 illustrates the occupancy rate, clock time (or critical path), and throughput results. The Virtex 6 xc6vlx240t-11156 FPGA by Xilinx was used. The first column, $N_{\mathrm{core}}$, indicates the number of cores implemented on FPGA. The second column, RN, displays the number of registers used. The third column, PR, displays the percentage of registers used regarding the total amount of available registers on the target FPGA (301440). The fourth and fifth columns, NLUT and PLUT, represent the amount of LUTs used in each implementation and the percentage of available LUTs used, respectively. The subsequent columns display the results of the clock time, denoted as $T_{s}$, measured in nanoseconds, and the throughput, denoted as $R_{s}$, measured in gigabits per second (Gbps). The final column reports the dynamic power consumption in watts. The rate of processing, denoted by $R_{s}$, for each *i*-th input message, can be calculated by

(31) $$R_{s}=\frac{K_{i}}{N_{\mathrm{clock}}}\times \frac{1}{T_{s}}\times N_{\mathrm{core}}=\frac{K_{i}\times N_{\mathrm{core}}}{N_{\mathrm{clock}}\times T_{s}}$$

where $N_{\mathrm{core}}$ represents the number of cores implemented on the FPGA (noting that more parallel cores yield higher throughput), and $N_{\mathrm{clock}}$ is the clock cycle count required by the FPGA to generate a single hash code [32]. In this implementation, the system processes several input block messages, with each *i*-th block input, $m_{i}$, comprising $K_{i}=64$ bytes (or 512 bits), and a clock cycle count $N_{\mathrm{clock}}=65$. Thus, for the values calculated in Table 1, the value of $R_{s}$ described in Equation (31) can be rewritten as

(32) $$R_{s}=\frac{512\times N_{\mathrm{core}}}{65\times T_{s}}.$$

Still, in Table 1, it is possible to see that the number of registers and LUTs used grows proportionally with the quantity of cores. When analyzing throughput, the difference between one and eight cores is approximately eight times. The implementation uses the concept of looping modules, keeping the hardware simple, but expands as more cores are added. Finally, it needs 64 iterations to generate the hash code and 1 iteration for the message to be available in the CO module (Figure 4). A maximum throughput of approximately 1.4 Gbps is observed when using $N_{\mathrm{core}}=16$ parallel cores on a single FPGA.

We observe a maximum throughput of around 1.4 Gbps when using $N_{\mathrm{core}}=16$ parallel cores on a single FPGA. This implementation model can generate hashes from a plain text password database. For example, 16 hashes are generated at 5.8 ms, resulting in around 2758 hashes per second (hash/s) for passwords up to 56 characters.

Table 2 shows the throughput, $R_{s}$, and speedup achieved over each reference. The first column lists the literature work with its respective year of publication. The second column shows the target FPGA, and in the third column, the achieved throughput. The last column shows the speedup values calculated between each throughput from the $R_{s}$ column and the 16-core architecture proposed in this work. The results presented are significant since, in the implementation here presented, the $N_{\mathrm{core}}=16$ cores of the structure from Figure 4 are executed entirely in parallel.

**Figure 8:**
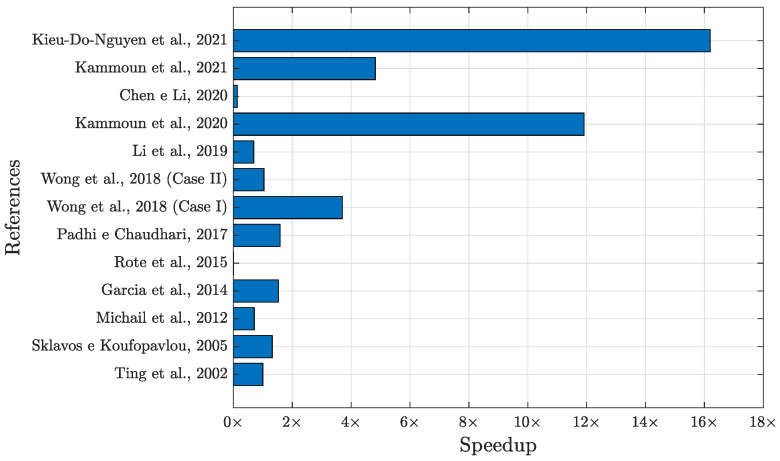
Speedup comparison between the proposal presented here and other works in the literature [3,21,30,31,32,33,35,36,37,38,57,58].

The results indicate that our implementation achieved a speedup of almost 5× when compared to [31], more than 3× concerning [58] (case I), 1.59× for [58] (case II) (the two cases in [58] use the architectural folding technique: case I is folded by 5 and has the lowest area cost; case II is folded by 2 and has a better balance between the area and throughput than case I), and 1.32× compared to [37]. In addition, it also achieved speedups of almost 12× compared to [32], more than 16× for [30], and a similar speed to [38]. The implementation of $N_{\mathrm{core}}=16$ fully parallel cores on Virtex-6 presented a speedup over almost all the studies presented in Table 2. The exceptions are [3,21,36,57]. Another way to visualize the results shown in Table 2 is through the bar graph shown in Figure 8, which indicates the same references as the first column of Table 2 on the y-axis and the speedup values on the *x*-axis. The red line on the *y*-axis in Figure 8 indicates a speedup of 1×. Values to the right of the red line represent lower throughput, indicating that our solution offers better speedup; values to the left are exceptions, which were listed earlier. The reasons for this are explained in the course of this section.

Table 3 shows additional information regarding the papers referenced here. The first and second columns are the same as in Table 2. The third column shows the number of slices present in each reference listed. Column four lists the operating clock frequencies in MHz, and column five, the throughput, $R_{s}$, in Gbps. The sixth column represents the throughput per slice (TPS), a metric used to measure the efficiency of each slice by throughput [36]. There is a better relationship between throughput and the amount of hardware used. Considering all the implementations from this table, the design proposed in this paper using $N_{\mathrm{core}}=16$ cores presented the result of 0.049 Mbps/slice.

Although the TPS values directly correlate with throughput and the total number of slices, the throughput calculation also considers the clock frequency, as indicated in Equation (31). Therefore, the relationship between these properties introduces a distinct method for calculating efficiency values.

Thus, considering the clock frequency in evaluating the implementation efficiency, this article proposes TPSF. TPSF compares TPS with clock frequency measured in Mbps/slice/MHz. We list the TPS results for the compared studies in the seventh column of Table 3. This method for calculating the efficiency of results has not been used previously in the literature related to implementations of hardware hash algorithms. However, using clock frequency to calculate efficiency in hardware development is directly related to power consumption, as explained in Section 6.2. With that in mind, when comparing the two references with the greater TPS values, [3,21], it is possible to verify that both have higher clock frequency values, by 24.83× and 15.45×, respectively. Thus, according to the new metric presented here, the TPSF, the implementation suggested in this article with $N_{\mathrm{core}}=1$ core presents the third-best and $N_{\mathrm{core}}=16$ cores presents the fifth-best value compared to the other related studies, with 0.0041 and 0.0044 Mbps/slice/MHz, respectively.

Despite not having the highest transactions per second per slice per MHz (TPSF) among all comparative proposals, our proposal could achieve higher throughput and TPSF if implemented with more cores. A fair comparison can be made by evaluating all architectures at the same clock frequency. For instance, if the architecture proposed in [21] operated at the same clock frequency as our proposal with one core (12.67 MHz), it would achieve a throughput of 6.487 Gbps, a TPS of 0.556 Mbps/slice, and a TPSF of 0.0439 Mbps/slice/MHz. The efficiency ratio would be 10× higher than our $N_{\mathrm{core}}=1$ core proposal at this frequency. However, more than 6× the amount of hardware resources (slices) are consumed.

**Figure 9:**
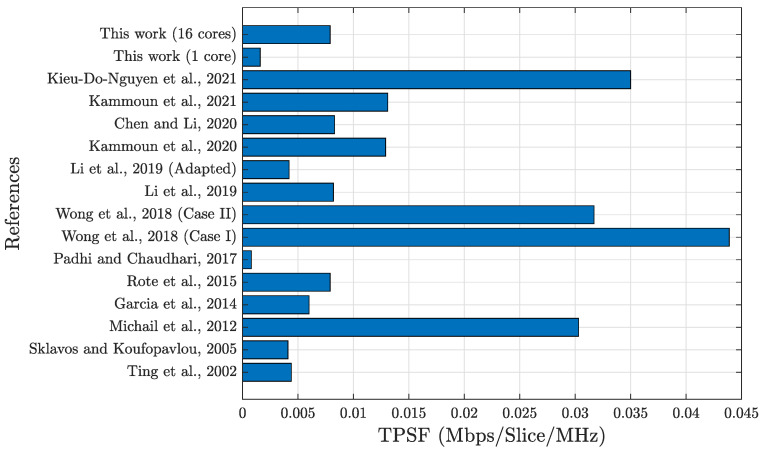
Comparing TPSFs of each literature reference [3,21,30,31,32,33,35,36,37,38,57,58].

The bar graph from Figure 9 illustrates each TPSF value from each reference graphically, complementing the information presented in Table 3.

### 6.2. Power Consumption

Table 4 shows the dynamic energy savings in relation to the dynamic power. According to [59], dynamic power (DP) can expressed as

(33) $$DP\propto N_{s}\times F_{\max}\times V_{\mathrm{dd}}^{2}$$

where $N_{s}$ is the number of elements (or slices), $F_{\max}$ is the maximum clock frequency, and $V_{\mathrm{dd}}^{2}$ means the supply voltage. Based on [60], the frequency is approximately proportional to the voltage at which a CMOS circuit can operate. Thereby, the dynamic power can be expressed as

(34) $$DP\propto N_{s}\times F_{\max}^{3}.$$

Based on Equation (34), the dynamic power saving can be expressed as

(35) $$S_{d}=\frac{N_{s}^{\mathrm{ref}}\times {\left(F_{\max}^{ref}\right)}^{3}}{N_{s}^{\mathrm{work}}\times {\left(F_{\max}^{\mathrm{work}}\right)}^{3}}$$

where $N_{s}^{ref}$ means the number of elements (slices), $F_{\max}^{ref}$ the maximum clock frequency of the reference works, and $N_{s}^{\mathrm{work}}$ and $F_{\mathrm{Max}}^{\mathrm{work}}$ are the number of elements (or slices) and the maximum clock frequency in this work, respectively [59].

Table 4 shows a comparison of the energy saving ratios, according to Equation (35), between the results of this work (implementations for $N_{\mathrm{core}}=1$ core and $N_{\mathrm{core}}=16$ cores) and all references in the cited literature (described in Section 2). The last two columns express the comparison between the implementation proposed here for one core and for sixteen cores in relation to the saving dynamic power (Sd).

This table presents the values from reference [21] recalculated with the clock frequency adjusted to 12.67 MHz (the same as the one-core proposal presented in this paper), as described in Table 3. In the Sd ($N_{\mathrm{core}}=1$ core) column, it is evident that the one-core proposal presented in this article saves more dynamic power than all other references. Moreover, the savings are considerable, reaching more than 4000× compared to references [36,57], and even more significant, at 9000×, when compared to [35]. Even though the one-core proposal uses more slices than most of the other proposed references, it still shows a significant difference in dynamic power consumption.

Regarding the freq. column from Table 4, the $N_{\mathrm{core}}=16$ cores full-parallel implementation, which allows the execution of 16 operations of the SHA-256 algorithm per clock cycle at 11.13 MHz, proved to be less than the others from the same column. Furthermore, this implementation also has the seventh highest throughput, at 1.4025 Gbps, which results in a dynamic power saving of 234.52× compared to [3], which has the highest throughput value, and 950.35× greater than [35]. It also shows savings of over 23× compared to [38], which has a similar throughput. Due to the non-linear relationship with clock frequency operation (Equation 35), the proposal presented here allows for impressive dynamic power savings compared to the other references, as shown in Table 4. When compared to the proposal with $N_{\mathrm{core}}=16$ cores presented here, there are gains compared to all references, with the exceptions of the proposals in [32] and the adapted one from [21], which presented values of 0.94× and 0.60×, respectively. However, the $N_{\mathrm{core}}=16$ cores proposal presented here has a throughput almost 12× greater than [32] and 4.6× less than [21]. Thus, the results presented indicate that the dynamic power savings of the implementation suggested here can reach considerable values, thereby validating the proposed hardware’s use in various IoT applications [59].

### 6.3. Blockchain in the IoT with FPGA

Based on Figure 1, Figure 2 and Figure 3, where each sensor’s data are treated as a transaction, it can be stated that each *p*-th IoT device must execute a Merkle tree structure every $T_{\mathrm{Markle}}^{p}$ seconds, where $T_{\mathrm{Marke}}^{p}$ must satisfy the following constraint

(36) $$T_{\mathrm{Markle}}^{p}\leq \min\left(T_{s_{1}}^{p},T_{s_{2}}^{p},\ldots ,T_{s_{k}}^{p},\ldots ,T_{s_{V_{p}}}^{p}\right)$$

where $T_{s_{k}}^{p}$ represents the acquisition time of the *k*-th sensor associated with the *p*-th IoT device. Each $T_{s_{k}}^{p}$ seconds each *k*-th sensor generates information $m_{i}$ with $K_{i}=64$ bytes (or 512 bits). Therefore, for each *p*-th device, the value of $T_{\mathrm{Markle}}^{p}$ can be calculated as

(37) $$T_{\mathrm{Markle}}^{p}=\left(\left(2\times V_{p}\right)-1\right)\times T_{\mathrm{Hash}}^{p}$$

where $T_{\mathrm{Hash}}^{p}$ is the time required to compute a hash in seconds associated with *p*-th device. This model ensures that the update of the Merkle tree on each IoT device does not exceed the shortest data acquisition interval among its connected sensors, thus maintaining the integrity and timeliness of the data processed for the blockchain.

Considering the number of cores associated with *p*-th device, $N_{\mathrm{core}}^{p}$, and acknowledging that the temporal dependence in the construction of the Merkle tree, implies that the computations for each subsequent level depends on the completion of the previous level. This means that while the presence of multiple processing cores ($N_{\mathrm{core}}^{p}>1$) can accelerate the computation of each level, the total parallel processing is constrained by the need for sequentiality between the levels. Given these implications, Equation (37) can be rewritten as

(38) $$T_{\mathrm{Markle}}^{p}=\sum_{i=1}^{\left\lceil \log_{2}\left(V_{p}\right)\right\rceil }\frac{2^{i-1}\times T_{\mathrm{Hash}}^{p}}{\min\left(N_{\mathrm{core}}^{p},2^{i-1}\right)}$$

where, based on Equations (31) and (32), $T_{\mathrm{Hash}}^{p}$ associated with the *p*-th IoT device can be expressed as

(39) $$T_{\mathrm{Hash}}^{p}=65\times T_{s}^{p}.$$

where $T_{s}^{p}$ is the FPGA clock time associated with the *p*-th IoT device.

Table 5 illustrates the computation times (in μs) required for updating Merkle tree structures under varying configurations. Each configuration is defined by the number of processing cores available, $N_{\mathrm{core}}^{p}$, and the number of sensors, $V_{p}$, of each IoT device. The computation time $T_{\mathrm{Marke}}^{p}$ is calculated based on Equation (38), which necessitates that each level of the tree must be completed before the next can begin. This table presents results for $N_{\mathrm{core}}^{p}$ values of 1 and 16 across different numbers of sensors, showing how parallel processing capabilities can significantly impact performance. The value $T_{s}^{p}$ indicates the base time to compute a single hash (associated with *p*-th device) and is expressed in nanoseconds (see Table 1). The data demonstrate how increasing the number of cores can decrease the time needed to update the Merkle tree, enhancing the system’s overall efficiency and responsiveness in a blockchain network.

The values of $T_{\mathrm{Markle}}^{p}$ presented in Table 5 indicate highly efficient processing times, particularly when considering real-world IoT sensor applications. Common IoT sensors, such as those measuring temperature, pressure, humidity, and others, typically have timing constraints that are considerably more lenient than the microseconds required to update the Merkle tree structure. For instance, most environmental sensors collect data in seconds or minutes, far exceeding the microsecond range needed for Merkle tree computation. These values demonstrate that the Merkle tree computation times, even for single-core configurations, are well suited to maintaining data integrity and security in real time without causing perceptible delays in device operations. Thus, it can be said that the values found in the table meet the constraint presented in Equation (36).

Furthermore, rapidly processing Merkle trees ensures that a broad range of IoT sensors can be efficiently integrated into blockchain-based systems without compromising performance or functionality. This efficiency is critical for critical applications where rapid response times and data security are paramount. Therefore, the results suggest that blockchain systems with the described hardware configuration can serve a wide array of IoT sensors, extending the application possibilities across various industries, from home automation to complex urban infrastructures and industrial environments, where diverse sensor types are essential for ongoing monitoring and control.

**Figure 10:**
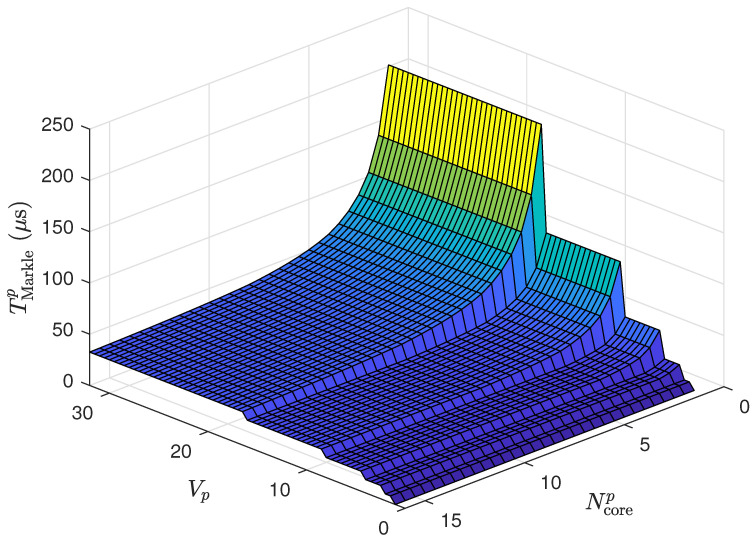
Graph of $T_{\mathrm{Markle}}^{p}$ across various sensor counts, $V_{p}$, and core numbers, $N_{\mathrm{core}}$, for the *p*-th IoT device.

Based on Table 1, the worst-case scenario in terms of critical time is for the case with $N_{\mathrm{core}}^{p}=16$ cores, which has a critical time of $T_{s}^{p}\approx 89$ ns. Assuming a clock time of $T_{s}^{p}=100$ ns for the *p*-th device, it is understood that this device can operate with any number of cores, $N_{\mathrm{core}}^{p}$, ranging from 1 to 16. Consequently, Figure 10 presents the graph of $T_{\mathrm{Markle}}^{p}$ for the *p*-th IoT device with various sensors (values of $V_{p}$ ranging from 1 to 32) and various numbers of cores (values of $N_{\mathrm{core}}^{p}$ from 1 to 16).

As depicted in Figure 10, as the number of sensors increases, so does the computation time, reflecting the added complexity of handling more data inputs when updating the Merkle tree. The steps or levels visible in the graph likely correspond to the incremental levels of the tree as more sensor data are assimilated. The number of cores exhibits a clear impact on performance, as the number of cores increases, the computation time decreases, highlighting the advantages of parallel processing. However, the benefit of adding more cores diminishes once the number of cores surpasses the parallelizable parts of the Merkle tree computation, as evidenced by the plateaus in the graph. The visual data suggest that increasing the number of processing cores up to a specific limit for IoT applications with high sensors can significantly improve computation times, which is crucial for real-time data processing and timely responses in IoT operations.

## 7. Conclusions

This work presents a hardware implementation of the SHA-256 algorithm on a Xilinx Virtex 6 xc6vlx240t-1ff1156 FPGA, adopting a parallel reuse approach for each iteration. This implementation offers flexibility in the number of cores used, ranging from one or a few for low-power applications to up to 16 for high-performance applications, capable of achieving a transfer rate of up to 1.4025 Gbps. The results are significant, as they are comparable to those found in the literature but exhibit a higher efficiency rate than those previously obtained. This research also introduces the TPSF metric, which measures throughput per slice per clock frequency, enabling a more equitable analysis given that each implementation utilizes clock frequency differently. From this perspective, the project proposed here with $N_{\mathrm{core}}=16$ cores achieved the fifth-best result among all compared studies. Dynamic power consumption was another variable compared in this study. The proposed implementation demonstrated substantial savings, up to 9614× for the $N_{\mathrm{core}}=1$ core implementation and approximately 950× for $N_{\mathrm{core}}=16$ cores. Therefore, it can be stated that the implementation proposed here performs exceptionally well in frequency comparison and is among the best in terms of dynamic power savings. Furthermore, this work details integrating the SHA-256 implementation into IoT applications using blockchain technology. It is demonstrated how the FPGA solution can swiftly handle the cryptographic operations necessary for securing data in blockchain networks, thus facilitating real-time data processing and enhancing IoT systems’ overall reliability and security. The FPGA-based SHA-256 implementation suits applications requiring high throughput and energy efficiency. Critical applications include secure data transmission in smart grids, real-time monitoring in healthcare, and authentication in financial transactions. The research targeted IoT scenarios needing robust security and efficient power use, such as smart cities and the Industrial IoT. These implementations ensure secure, high-speed data processing and minimal energy consumption, addressing critical issues in data integrity and energy efficiency in IoT devices.

## Figures and Tables

**Table 1:** Results regarding occupancy, clock time, and throughput for various cores.

$N_{\mathrm{core}}$	RN	PR	NLUT	PLUT	$T_{s}$	$R_{s}$	DP
		(%)		(%)	(ns)	(Gbps)	(Watts)
1	794	0.26	6730	4.47	78.957	0.0998	0.042
2	1582	0.52	13,482	8.95	79.727	0.1976	0.082
4	3158	1.05	26,933	17.87	81.808	0.3851	0.145
8	6313	2.09	53,691	35.62	89.037	0.7077	0.255
16	12,618	4.19	107,609	71.40	89.864	1.4025	0.503

**Table 2:** Throughput comparison with other referenced works.

Reference	TargetFPGA	$R_{s}$ (Gbps)	Speedup (Comparing This Work for $N_{core}=16$ Cores)
[30] 2002	Virtex XCV300E−8	0.087	16.12×
[31] 2005	Virtex v200pq240	0.291	4.82×
[3] 2012	Xilinx Virtex6	11.008	0.13×
[32] 2014	Xilinx Virtex5	0.1178	11.91×
[57] 2015	Xilinx Virtex6	2.041	0.69×
[33] 2017	Xilinx Virtex4	1.3449	1.04×
[58] 2018 (Case I)	Xilinx Virtex4	0.379	3.70×
[58] 2018 (Case II)	Xilinx Virtex4	0.881	1.59×
[21] 2019	Xilinx Virtex6	141.517	0.01×
[35] 2020	Zynq−7000 XC7Z020	0.917	1.53×
[36] 2020	Xilinx Virtex4	1.984	0.71×
[37] 2021	Zynq−7000 XC7Z020	1.063	1.32×
[38] 2021	Xilinx Virtex4	1.404	1.0×

**Table 3:** Comparative table between related publications and those proposed using the TPS and TPSF approach.

Reference	TargetFPGA	Slices	Freq. (MHz)	$R_{s}$ (Gbps)	TPS (Mbps/Slice)	TPSF (Mbps/Slice/MHz)
[30] 2002	Virtex XCV300E−8	1261	88	0.087	0.699	0.0079
[31] 2005	Virtex v200pq240	2384	74	0.291	0.122	0.0016
[3] 2012	Xilinx Virtex6	1831	172	11.008	6.012	0.0350
[32] 2014	Xilinx Virtex5	139	64.45	0.1178	0.847	0.0131
[57] 2015	Xilinx Virtex6	905	271	2.041	2.255	0.0083
[33] 2017	Xilinx Virtex4	610	170.75	1.3449	2.204	0.0129
[58] 2018(CaseI)	Xilinx Virtex4	382	238	0.379	0.99	0.0042
[58] 2018(CaseII)	Xilinx Virtex4	485	222	0.881	1.82	0.0082
[21] 2019	Xilinx Virtex6	11,660	276.4	141.517	8.768	0.0317
[21] 2019—Adapted	Xilinx Virtex6	11,660	12.67	6.487	0.556	0.0439
[35] 2020	Zynq−7000 XC7Z020	6367	181	0.917	0.144	0.0008
[36] 2020	Xilinx Virtex4	979	255.7	1.984	2.027	0.0079
[37] 2021	Zynq−7000 XC7Z020	1305	135	1.063	0.815	0.0060
[38] 2021	Zynq−7000 XC7Z020	327	141.84	1.404	4.294	0.0303
Thiswork ($N_{core}=1$ core)	Xilinx Virtex6	1933	12.67	0.0998	0.052	0.0041
Thiswork ($N_{core}=16$ cores)	Xilinx Virtex6	28,830	11.13	1.4025	0.049	0.0044

**Table 4:** Comparative of dynamic power saving with other papers.

Reference	TargetFPGA	Slices	Freq. (MHz)	$R_{s}$ (Gbps)	TPS (Mbps/Slice)	$S_{d}$ $N_{core}=1$	$S_{d}$ $N_{core}=16$
[30] 2002	Virtex XCV300E−8	1261	88	0.087	0.699	218,83×	21.63×
[31] 2005	Virtex v200pq240	2384	74	0.291	0.122	246.00×	24.32×
[3] 2012	Xilinx Virtex6	1831	172	11.008	6.012	2372.54×	234.52×
[32] 2014	Xilinx Virtex5	139	64.45	0.1178	0.847	9.48×	0.94×
[57] 2015	Xilinx Virtex6	905	271	2.041	2.255	4586.66×	453.39×
[33] 2017	Xilinx Virtex4	610	170.75	1.3449	2.204	773.31×	76.44×
[58] 2018 (Case I)	Xilinx Virtex4	382	238	0.379	0.99	1311.40×	129.63×
[58] 2018 (Case II)	Xilinx Virtex4	485	222	0.881	1.82	1351.27×	133.57×
[21] 2019—Adapted	Xilinx Virtex6	11,660	12.67	6.487	0.556	6.04×	0.60×
[35] 2020	Zynq−7000 XC7Z020	6367	181	0.917	0.144	9614.14×	950.35×
[36] 2020	Xilinx Virtex4	979	255.7	1.984	2.027	4167.88×	411.99×
[37] 2021	Zynq−7000 XC7Z020	1305	135	1.063	0.815	817.62×	80.82×
[38] 2021	Xilinx xc7a200t	327	141.84	1.404	4.294	237.62×	23.49×
This work ($N_{core}=1$)	Xilinx Virtex6	1993	12.67	0.0998	0.052	−	−
This work ($N_{core}=16$)	Xilinx Virtex6	12,618	11.13	1.4025	0.049	−	−

**Table 5:** Values of $T_{\mathrm{Markle}}^{p}$ for different $N_{\mathrm{core}}^{p}$ and $V_{p}$ associated with *p*-th IoT device.

$N_{\mathrm{core}}^{p}$	$T_{s}^{p}$ in Nano Sec (ns)	$V_{p}$	$T_{\mathrm{Markle}}^{p}$ in μs
1	78.957	4	15.397
16	89.864	4	11.682
1	78.957	8	35.925
16	89.864	8	17.523
1	78.957	16	76.983
16	89.864	16	23.365

## Data Availability

Data are contained within the article.
